# Nationwide Geographical and Temporal Distribution of Tick-Borne Diseases in Korean Water Deer (*Hydropotes inermis argyropus*)

**DOI:** 10.3390/ani15101499

**Published:** 2025-05-21

**Authors:** Beoul Kim, Su-Jin Chae, You-Jeong Lee, Haksub Shin, Sunmin Kwak, Hyesung Jeong, Suwoong Lee, Dongmi Kwak, Min-Goo Seo

**Affiliations:** 1 College of Veterinary Medicine & Institute for Veterinary Biomedical Science, Kyungpook National University, 80 Daehak-ro, Buk-gu, Daegu 41566, Republic of Korea; kbjjhnm@naver.com (B.K.); wowgirlsgood@naver.com (Y.-J.L.); dmkwak@knu.ac.kr (D.K.); 2 Wildlife Disease Research Team, National Institute of Wildlife Disease Control and Prevention, 1 Songam-gil, Gwangju 62407, Republic of Korea; chsj1215@korea.kr (S.-J.C.); tgs00089@korea.kr (H.S.); sunmin3416@naver.com (S.K.); halley@korea.kr (H.J.); hoffman@korea.kr (S.L.)

**Keywords:** tick-borne diseases, Korean water deer, *Anaplasma* spp., *Borrelia* spp., *Theileria* spp.

## Abstract

Ticks are major vectors of numerous pathogens, and their public health significance is increasing due to climate change and the expanding involvement of wildlife hosts. In Korea, the Korean water deer (KWD, *Hydropotes inermis argyropus*) is widely distributed and considered a key wild host for ticks, potentially serving as a reservoir for diverse tick-borne diseases (TBDs). To examine the geographical and temporal distribution of TBDs in this species, 1035 spleen samples were collected from KWDs across 12 regions between April and November 2023. Overall, 93.6% (969) of samples were positive for at least one pathogen. Five *Anaplasma* species were identified: *A. phagocytophilum*, *A. phagocytophilum*-like A (APLA), APLB, *A. bovis*, and *A. capra*. In addition, *Borrelia theileri* and three *Theileria* species—*T. capreoli, T. cervi*, and *T. luwenshuni*—were detected. Notably, this study presents the first global detection of APLA, APLB, and *B. theileri* in KWDs. Statistically significant regional and seasonal differences in prevalence were observed, with higher detection rates in northern regions and during autumn, suggesting that environmental and ecological factors influence transmission dynamics. These findings highlight the role of KWD as a reservoir host for multiple TBDs and underscore the importance of wildlife-based surveillance for assessing zoonotic disease risks.

## Simple Summary

Tick-borne diseases are becoming more common around the world due to climate change and increasing contact between wildlife and humans. In Korea, the Korean water deer is a widely distributed wild animal that often carries ticks. These ticks can spread diseases that may harm both animals and people. To better understand this risk, we conducted a nationwide study between April and November 2023, collecting spleen samples from over 1000 Korean water deer across 12 regions. We found that over 90% of the deer were infected with at least one disease-causing organism. Several types of bacteria and parasites were identified, including some that had never before been found in this species anywhere in the world. The most common were *Anaplasma phagocytophilum* and *Theileria luwenshuni*, which can cause serious illness in animals and have potential implications for human health. We also found that infections were more common in northern regions and during autumn, suggesting that weather and environmental factors affect disease spread. This research shows that Korean water deer may play an important role in the circulation of tick-borne diseases in Korea and highlights the need for wildlife monitoring to help protect public and animal health.

## 1. Introduction

Ecological changes such as climate change, increased wildlife involvement as hosts, and declining environmental biodiversity have contributed to a rise in tick infestations among animals [1]. These shifts are reshaping the epidemiology and geographic distribution of tick infestations, leading to a corresponding increase in tick-borne diseases (TBDs) [2]. Ticks are capable of transmitting multiple zoonotic pathogens concurrently, and their ability to attach to mobile hosts enables rapid and widespread geographic dissemination. As a result, several TBDs are no longer restricted to specific regions and may potentially emerge in new areas globally [3]. The wide distribution of ticks and their role as vectors of diverse zoonotic pathogens underscore a growing public health concern and reinforce the need for sustained surveillance and proactive control measures.

In Korea, the Korean water deer (*Hydropotes inermis argyropus*) is a key wildlife species implicated in the transmission of TBDs. Despite being classified as a globally vulnerable species on the International Union for Conservation of Nature Red List due to population declines from poaching and habitat destruction [4], the Korean water deer remains widely distributed in the wild across Korea [5]. This species is frequently infested with ticks that serve as vectors for numerous zoonotic pathogens, thereby raising concerns about their potential role in zoonotic transmission. Among the tick-borne pathogens identified in Korean water deer, *Anaplasma* spp. are obligate intracellular bacteria that infect white blood cells and can cause anaplasmosis, characterized by fever, anorexia, and leukopenia in animals and humans [6]. *Borrelia* spp., including *B. theileri*, are spirochetes responsible for relapsing fever or Lyme-like syndromes [7]. *Theileria* spp. are protozoan parasites of the blood and lymphoid tissues, capable of causing febrile illness and lymphadenopathy in ungulates [8]. These pathogens complete parts of their life cycles within both vertebrate hosts and tick vectors, highlighting the ecological complexity of TBD transmission. Although clinical symptoms in wild deer are often subclinical or unreported, these pathogens pose potential risks for cross-species transmission and human infection.

In particular, Korean studies have detected *Anaplasma* spp., *Babesia* spp., and *Coxiella* spp. in Korean water deer [9], and reported the presence of *Ehrlichia* spp., *Theileria* spp. [10], and the Severe Fever with Thrombocytopenia Syndrome (SFTS) virus [11]. Additionally, ticks parasitizing Korean water deer have been found to harbor various pathogens, including *Anaplasma* spp., *Bartonella* spp., *Rickettsia* spp. [12], *Borrelia* spp. [13], and other strains of *Ehrlichia* spp., *Rickettsia* spp., and *Anaplasma* spp. [14].

These findings suggest that both the Korean water deer and their ectoparasitic ticks may function as significant reservoirs and vectors of zoonotic diseases, underscoring the importance of continued surveillance. Given the dual risk associated with the host and its ectoparasites, it is critical to investigate not only the pathogens infecting the deer directly but also those carried by the attached ticks. Therefore, the present study aims to systematically examine the geographical and temporal trends of TBDs in Korea by analyzing spleen samples from Korean water deer collected nationwide. We screened for seven pathogens: *Anaplasma* spp., *Babesia* spp., *Borrelia* spp., and *Theileria* spp.—all previously reported in Korea—as well as three emerging viruses of concern in neighboring countries that have not yet been detected domestically: Tick-borne encephalitis virus (TBEV), OZ virus (OZV), and Yezo virus (YezoV).

## 2. Materials and Methods

### 2.1. Ethical Approval

All spleen samples were obtained from Korean water deer captured in the wild between April and November 2023. Sampling sites were selected based on regional variations in the incidence of human SFTS cases, encompassing both high-incidence and control areas with relatively low incidence. Within these predefined regions, deer were captured by licensed hunters commissioned by the National Institute of Wildlife Disease Control and Prevention (NIWDC), operating under the Ministry of Environment, Republic of Korea. The collected spleen tissues were subsequently provided by the NIWDC for research purposes. All experimental procedures were approved by the Institutional Animal Care and Use Committee of Kyungpook National University (Approval No. KNU 2024-0407).

### 2.2. Sample Collection

**Figure 1:**
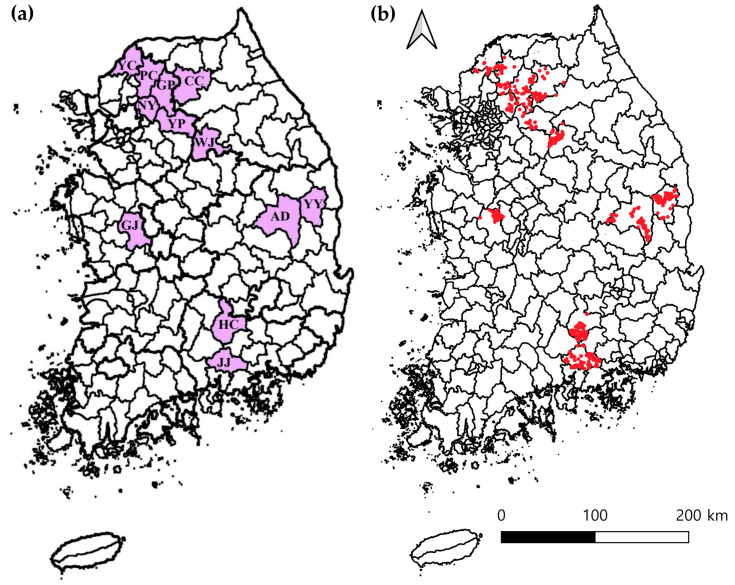
Sampling locations of Korean water deer across Korea. (**a**) Regional distribution of the 12 collection sites, categorized into northern, central, and southern regions. The northern region includes Wonju (WJ), Chuncheon (CC), Gapyeong (GP), Namyangju (NY), Yangpyeong (YP), Yeoncheon (YC), and Pocheon (PC). The central region includes Andong (AD), Yeongyang (YY), and Gongju (GJ), while the southern region includes Jinju (JJ) and Hapcheon (HC). (**b**) Geospatial distribution of GPS coordinates representing all 1035 Korean water deer individuals sampled between April and November 2023. Each red dot represents a single sampling location.

Between April and November 2023, spleen tissue samples were collected from a total of 1035 Korean water deer across 12 regions of Korea. The northern region included Wonju (n = 134), Chuncheon (n = 72), Gapyeong (n = 60), Namyangju (n = 56), Yangpyeong (n = 31), Yeoncheon (n = 60), and Pocheon (n = 85). In the central region, samples were collected from Andong (n = 125), Yeongyang (n = 78), and Gongju (n = 104). The southern region included Jinju (n = 115) and Hapcheon (n = 115) (Figure 1a). Geographic coordinates were recorded for each sampled individual, and a spatial map showing all 1035 sampling locations was generated (Figure 1b). These data were collected on-site using handheld GPS devices at the time of sampling. The GPS coordinates and collection dates of all 1035 sampled individuals are provided in Supplementary Table S1.

By month, samples were obtained from 56 Korean water deer in April, 69 in May, 83 in June, 89 in July, 114 in August, 207 in September, 250 in October, and 167 in November.

### 2.3. Nucleic Acids Extraction and Processing

For gene extraction, spleen tissues from Korean water deer were homogenized using the Precellys CK28-R Lysing Kit (bead tube for hard tissue homogenization; Bertin Technologies, Bretonneux, France) in combination with the Precellys Evolution Homogenizer (Bertin Technologies). Approximately 10 mg of spleen tissue was used per sample. For tissue lysis, 710 µL of lysis buffer was prepared by mixing 700 µL of DLD buffer (lysis agent) with 10 µL of β-mercaptoethanol (reducing agent). The entire volume was applied to each tissue sample for nucleic acid extraction. Genomic DNA and RNA were simultaneously extracted using the Clear-S™ Quick DNA Extraction Kit (Invirustech, Gwangju, Republic of Korea), following a modified protocol optimized for spleen samples from Korean water deer. Detailed steps of the modified protocol are provided in Appendix A.

### 2.4. Detection of Diseases Using Real-Time PCR

Real-time PCR was employed to detect seven TBDs. Pathogen detection was carried out using commercially available real-time PCR kits in accordance with the manufacturers’ protocols. The “SB-Plex™ Tickborne System 1” (SNB, Gwangmyeong, Republic of Korea) was used to detect *Anaplasma* spp., *Borrelia* spp., and *Theileria* spp., targeting the 23S rDNA region for *Borrelia* spp. and the 16S rDNA region for both *Anaplasma* spp. and *Theileria* spp. The primers for *Borrelia* spp. were designed to detect a broad range of zoonotic species within the *Borrelia burgdorferi* sensu lato complex but were not specific to *B. burgdorferi* sensu stricto. The “SB-Plex™ YeV/OzV/TBEV qPCR Kit” (SNB) was used to detect YezoV, OZV, and TBEV, targeting the M segment for YezoV and OZV, and the 5′ untranslated region (UTR) for TBEV. For the detection of *Babesia* spp., the “AniQvet *Babesia* qPCR Kit“ (Koreagene, Seoul, Republic of Korea) was utilized, targeting the 18S rDNA region as per the manufacturer’s instructions.

### 2.5. Detection of Diseases Using Conventional PCR

Conventional PCR was performed to validate the positive findings obtained through real-time PCR and confirm the sequence identity of each detected pathogen. Species-specific primers described in previous studies were used to amplify target gene regions of *Anaplasma* spp. [15], *Borrelia* spp. [16], and *Theileria*/*Babesia* spp. [17]. For the detection of bacterial and parasitic pathogens, the AccuPower^®^ PCR Premix kit (Bioneer, Daejeon, Republic of Korea) was utilized. For viral pathogens, amplification was performed using the AccuPower^®^ RT-PCR Premix kit (Bioneer). To ensure the accuracy and reproducibility of PCR results, both positive and negative controls were included in all assays.

### 2.6. Genetic Sequencing and Phylogenetic Study

PCR amplicons confirmed as positive for the target pathogens were submitted to Macrogen (Seoul, Republic of Korea) for nucleotide sequencing along with the corresponding primers. All samples confirmed as positive were subjected to sequencing analysis, and in cases where identical sequences were obtained, a single representative sequence was selected for further analysis. The resulting sequences were then compared with reference sequences available in the NCBI GenBank database using the BLAST algorithm (BLAST+ v. 2.14.1) provided by NCBI against the nt (nucleotide collection) database to facilitate molecular genetic analysis. Sequence alignment was conducted using CLUSTAL Omega (v. 1.2.1, Bioweb, Ferndale, WA, USA), and redundant sequences were eliminated using BioEdit (v. 7.2.5). Regions exhibiting ambiguous alignments or containing gaps were excluded prior to phylogenetic analysis. Phylogenetic relationships were inferred using MEGA software (v. 6.0) [18], employing the Kimura two-parameter distance model [19] and the maximum likelihood method, with bootstrap support calculated from 1000 replicates. Only values ≥ 70% were shown on the phylogenetic trees.

### 2.7. Statistical Examination

All statistical analyses were performed using GraphPad Prism (v. 5.04, GraphPad Software Inc., La Jolla, CA, USA). Pearson’s chi-square test was applied to contingency tables involving more than two variables. A *p*-value of ≤ 0.05 was considered statistically significant, and 95% confidence intervals (CIs) were calculated for all estimates.

### 2.8. GenBank Accession Numbers

*Anaplasma phagocytophilum* sequences generated in this study were deposited in GenBank under the following accession numbers: PV362647–PV362649, PV362652, PV362654, PV362657, PV362659, PV362663–PV362670, PV362672, PV362674, PV362675, PV362678, PV362682, and PV362685. APLA sequences generated in this study were deposited in GenBank under the following accession numbers: PV362660, PV362661, PV362653, PV362673, PV362681, PV362683, PV362684, and PV362687. APLB sequences generated in this study were deposited in GenBank under the following accession numbers: PV362643, PV362644, PV362645, PV362646, PV362650, PV362662, PV362676, PV362677, and PV362686. *A. bovis* sequences generated in this study were deposited in GenBank under the following accession numbers: PV362679 and PV362680. *A. capra* sequences generated in this study were deposited in GenBank under the following accession numbers: PV362651, PV362655, PV362656, PV362658, and PV362671.

*Borrelia theileri* sequences generated in this study were deposited in GenBank under the following accession numbers: PV389464–PV389471.

*Theileria capreoli* sequences generated in this study were deposited in GenBank under the following accession numbers: PV436602–PV436606, PV436611, PV436613–PV436619, PV436622, PV436623, PV436626, PV436627, PV436629, PV436633, PV436634, and PV436636. *T. cervi* sequences generated in this study were deposited in GenBank under the following accession numbers: PV436624, PV436625, and PV436631. *T. luwenshuni* sequences generated in this study were deposited in GenBank under the following accession numbers: PV436601, PV436607-PV436610, PV436612, PV436620, PV436621, PV436628, PV436630, PV436632, PV436635, and PV436637.

## 3. Results

### 3.1. Prevalence Analysis Using Real-Time PCR

Real-time PCR analysis detected four tick-borne pathogens—*Anaplasma* spp., *Babesia* spp., *Borrelia* spp., and *Theileria* spp.—in Korean water deer samples. *Anaplasma* spp. and *Theileria* spp. showed high detection frequencies, while *Babesia* spp. and *Borrelia* spp. were identified at lower rates. No positive results were observed for TBEV, the OZ virus, or the Yezo virus in any of the 1035 samples tested. Detailed detection rates and confidence intervals are presented in Table 1.

### 3.2. Prevalence Analysis Using Conventional PCR

Conventional PCR analysis identified 9 TBDs, with 969 out of 1035 samples testing positive for at least one pathogen, corresponding to an overall positivity rate of 93.6% (95% CI 92.1–95.1) (Table 2). *Anaplasma* spp. were classified into five distinct species: *A. phagocytophilum* (804 samples; 77.7%; 95% CI 75.1–80.2), *A. phagocytophilum*-like A (APLA; 9 samples; 0.9%; 95% CI 0.3–1.4), *A. phagocytophilum*-like B (APLB; 59 samples; 5.7%; 95% CI 4.3–7.1), *A. bovis* (26 samples; 2.5%; 95% CI 1.6–3.5), and *A. capra* (8 samples; 0.8%; 95% CI 0.2–1.3). *Borrelia* spp. was detected solely as *B. theileri* (69 samples; 6.7%; 95% CI 5.1–8.2). Although *Babesia* and *Theileria* spp. were amplified using a common primer set, only *Theileria* spp. were identified. These were further classified into *T. capreoli* (284 samples; 27.4%; 95% CI 24.7–30.2), *T. cervi* (6 samples; 0.6%; 95% CI 0.1–1.0), and *T. luwenshuni* (628 samples; 60.7%; 95% CI 57.7–63.7).

**Figure 2:**
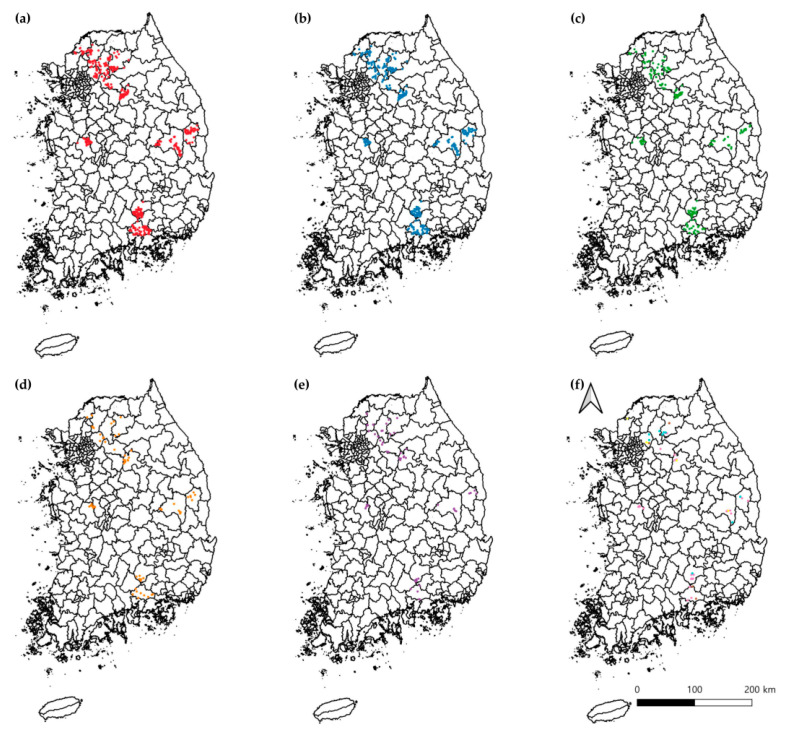
Spatial distribution of Korean water deer individuals testing positive for tick-borne pathogens detected by conventional PCR. Each colored dot represents an individual infected with a specific pathogen. (**a**) *Anaplasma phagocytophilum* (red), (**b**) *Theileria luwenshuni* (blue), (**c**) *Theileria capreoli* (green), (**d**) *Borrelia theileri* (orange), (**e**) *Anaplasma phagocytophilum*-like B (purple), and (**f**) Combined distribution of *A. phagocytophilum*-like A (teal), *A. bovis* (pink), *A. capra* (brown), and *T. cervi* (yellow) due to their relatively low prevalence. Each map displays the GPS coordinates of infected individuals, providing spatial insight into the regional clustering and distribution patterns of each pathogen.

Spatial distribution maps were generated to visualize the geographic location of individuals that tested positive for each pathogen (Figure 2). *A. phagocytophilum*-positive samples were widely distributed across the country, particularly concentrated in the northern region, including Namyangju (Figure 2a). Similarly, a high regional density of *T. luwenshuni*-positive samples was observed in Chuncheon (Figure 2b). *T. capreoli* (Figure 2c) was spatially concentrated in Yangpyeong, while *B. theileri*-positive individuals were more sporadically distributed but with notable clustering in central regions (Figure 2d). APLB infections were mostly detected in northern sites, especially in Yangpyeong (Figure 2e). Due to the relatively low prevalence of APLA, *A. bovis, A. capra*, and *T. cervi*, these pathogens were grouped and are visualized collectively in Figure 2f.

By region, the highest positivity rates were observed in Namyangju (56/56; 100%) and Yangpyeong (31/31; 100%). The regional comparison revealed a statistically significant difference in positivity rates (χ^2^ = 92.305, df = 11, *p* < 0.0001) (Table 2). Pathogen-specific analyses showed the highest prevalence of *A. phagocytophilum* in Namyangju (89.3%; 95% CI 81.2–97.4; χ² = 36.576, df = 11, *p* = 0.0001) and *A. capra* in Chuncheon (5.6%; 95% CI 0.3–10.8; χ^2^ = 29.948, df = 11, *p* = 0.0016), both reaching statistical significance. Similarly, *T. capreoli* was most prevalent in Yangpyeong (54.8%; 95% CI 37.3–72.4; χ^2^ = 52.410, df = 11, *p* < 0.0001), *T. cervi* in Namyangju (5.4%; 95% CI 0–11.3; χ^2^ = 27.425, df = 11, *p* = 0.0400), and *T. luwenshuni* in Chuncheon (73.6%; 95% CI 63.4–83.8; χ^2^ = 21.561, df = 11, *p* = 0.0280), all of which also demonstrated statistically significant regional variation. These statistical results were in accordance with the spatial patterns observed in Figure 2.

**Figure 3:**
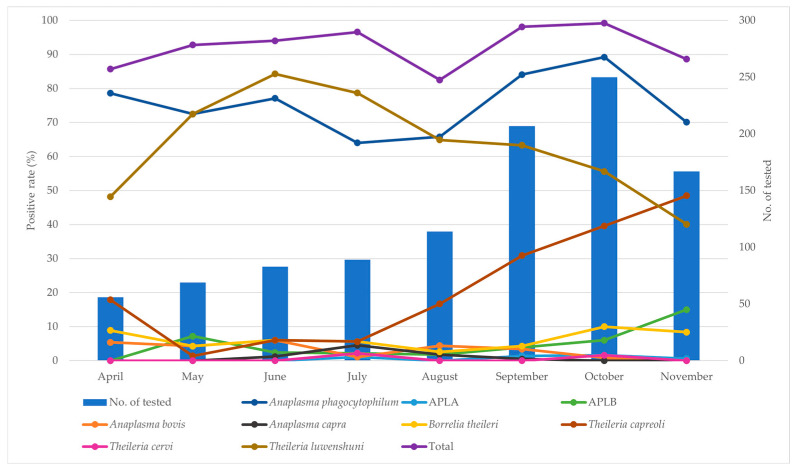
Temporal distribution of Korean water deer captured and the monthly infection rates of nine pathogens detected from April to November 2023 in Korea. The line graph represents pathogen infection rates. Abbreviations: APLA, *Anaplasma phagocytophilum*-like A; APLB, *Anaplasma phagocytophilum*-like B.

By season, the overall positivity rate peaked in October (248/250; 99.2%), with a significant difference observed across seasons (χ^2^ = 57.998, df = 7, *p* < 0.0001) (Table 3 and Figure 3). Season-specific analysis indicated the highest prevalence of *A. phagocytophilum* in October (89.2%; 95% CI 85.4–93.0; χ^2^ = 49.551, df = 7, *p* < 0.0001), APLB in November (15.0%; 95% CI 9.6–20.4; χ^2^ = 38.674, df = 7, *p* < 0.0001), *A. bovis* in June (6.0%; 95% CI 0.9–11.1; χ^2^ = 17.251, df = 7, *p* = 0.0158), and *A. capra* in July (4.5%; 95% CI 0.2–8.8; χ^2^ = 22.153, df = 7, *p* = 0.0024), all showing statistically significant seasonal patterns. Among *Theileria* species, the prevalence of *T. capreoli* was highest in November (48.5%; 95% CI 40.9–56.1; χ^2^ = 130.080, df = 7, *p* < 0.0001), while *T. luwenshuni* was most prevalent in June (84.3%; 95% CI 76.5–92.2; χ^2^ = 72.914, df = 7, *p* < 0.0001), both with significant seasonal variation.

### 3.3. Molecular and Phylogenetic Analyses

**Figure 4:**
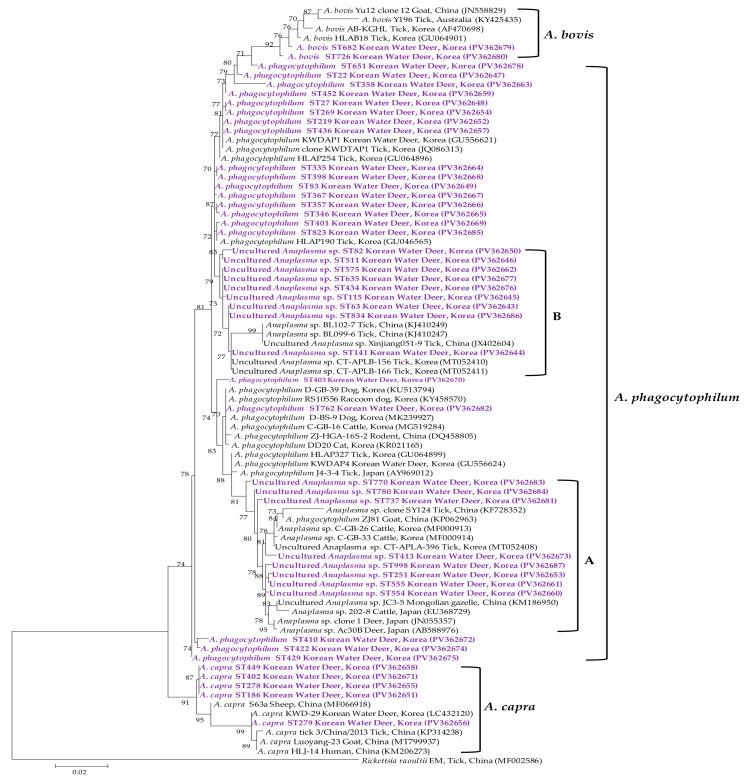
Phylogenetic tree constructed using the maximum likelihood method based on *Anaplasma* spp. 16S rRNA gene sequences. Sequences generated in this study are indicated in purple font. GenBank accession numbers for reference sequences are shown next to each label. Bootstrap values from 1000 replicates are shown at the respective branches, and the scale bar indicates genetic distance. Sequences labeled ‘A’ and ‘B’ represent *A. phagocytophilum*-like A (APLA) and B (APLB), respectively.

Phylogenetic analysis of the 16S rRNA sequences of *Anaplasma* spp. revealed classification into *A. phagocytophilum*, APLA, APLB, *A. bovis*, and *A. capra* (Figure 4). The 21 representative *A. phagocytophilum* sequences exhibited 98.3–99.9% identity with one another and 98.3–100% identity with previously deposited *A. phagocytophilum* isolates in GenBank. The eight representative APLA sequences showed 98.5–99.5% identity with one another and 97.4–99.5% identity with reference APLA isolates in GenBank. Similarly, the nine representative APLB sequences demonstrated 98.9–100% identity among themselves and 97.5–100% identity with previously reported APLB isolates. The two representative *A. bovis* sequences displayed 98.4% identity with each other and 96.0–98.9% identity with GenBank-deposited *A. bovis* isolates. The five representative *A. capra* sequences shared 97.6–99.8% identity with one another and 97.6–100% identity with *A. capra* sequences in GenBank.

**Figure 5:**
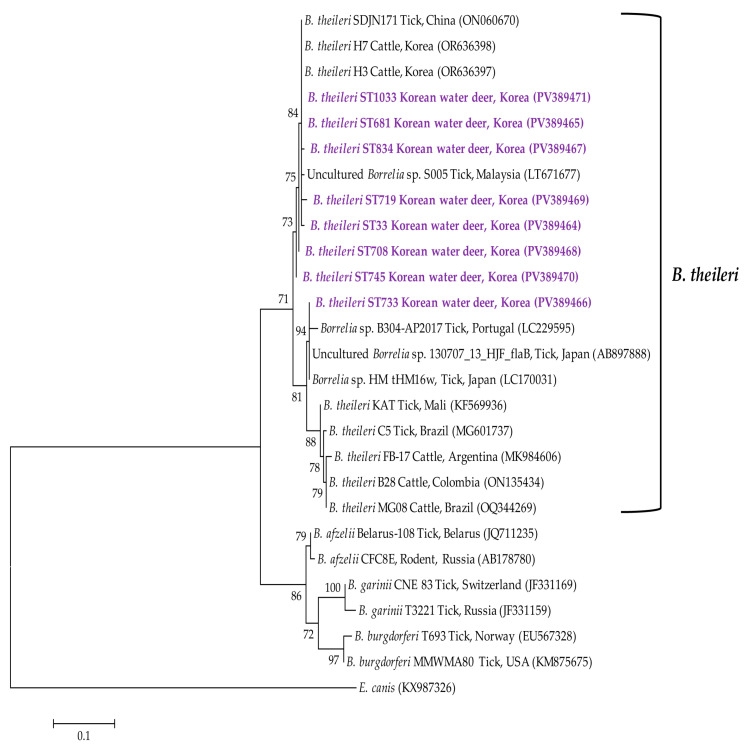
Phylogenetic tree constructed using the maximum likelihood method based on *Borrelia* spp. flagellin B gene sequences. Sequences generated in this study are indicated in purple font. GenBank accession numbers for reference sequences are shown next to each label. Bootstrap values from 1000 replicates are shown at the respective branches, and the scale bar indicates genetic distance.

Phylogenetic analysis of the *Borrelia* spp. flagellin B gene sequence identified the organism as *B. theileri* (Figure 5). The eight representative *B. theileri* sequences exhibited 95.1–99.7% identity with each other and 95.1–100% identity with *B. theileri* isolates previously deposited in GenBank.

**Figure 6:**
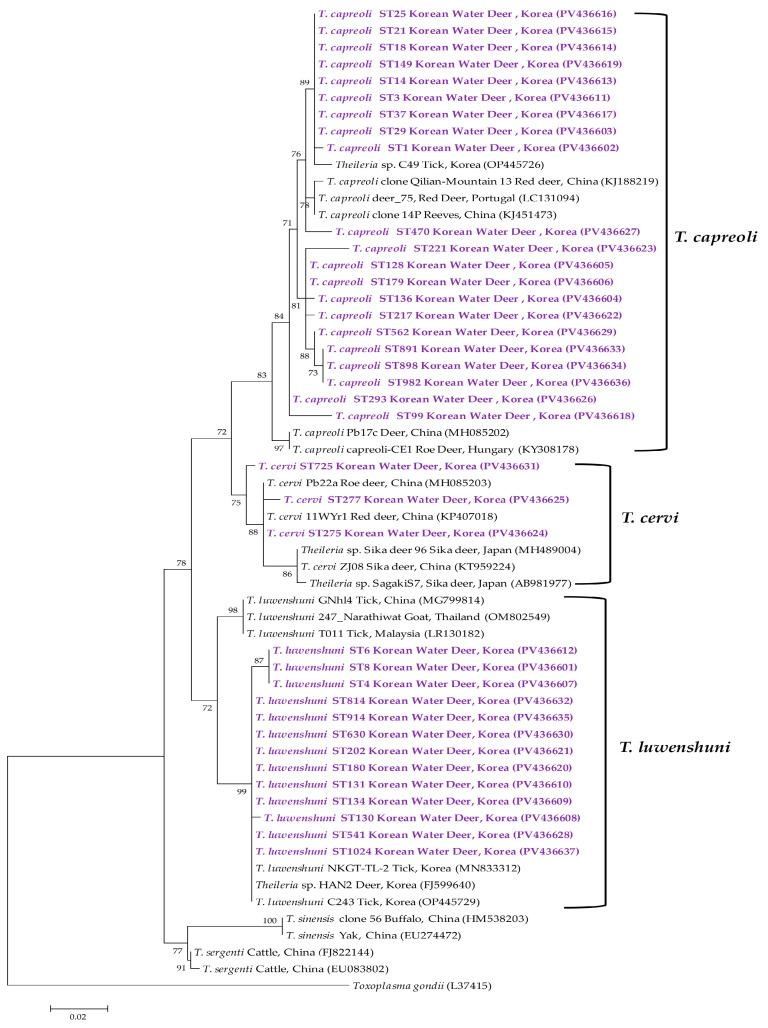
Phylogenetic tree constructed using the maximum likelihood method based on *Theileria* spp. 18S rRNA gene sequences. Sequences generated in this study are indicated in purple font. GenBank accession numbers for reference sequences are shown next to each label. Bootstrap values from 1000 replicates are shown at the respective branches, and the scale bar indicates genetic distance.

Analysis of the 18S rRNA sequences of *Theileria* spp. indicated classification into *T. capreoli*, *T. cervi*, and *T. luwenshuni* (Figure 6). The 21 representative *T. capreoli* sequences shared 93.1–93.6% identity with one another and 94.9–97.2% identity with corresponding GenBank isolates. The three representative *T. cervi* sequences exhibited 97.7–99.5% identity among themselves and 94.4–98.5% identity with GenBank *T. cervi* sequences. Finally, the 13 representative *T. luwenshuni* sequences showed 96.4–100% identity with each other and 92.6–97.8% identity with previously deposited *T. luwenshuni* isolates.

## 4. Discussion

In recent years, the spectrum of TBDs affecting both livestock and humans has expanded, with increasing attention focused on zoonotic TBDs such as anaplasmosis, babesiosis, ehrlichiosis, and Lyme borreliosis in both medical and veterinary contexts [2]. Beyond the pathogens identified in the present study, tick-borne viral infections have also garnered growing concern. For example, TBEV is considered endemic in several regions, including Siberia, the Russian Far East, China, and Japan [20]. Human and wildlife infections with OZV were reported in Japan between 2013 and 2019 [21] and YezoV was first identified in humans in Japan in 2019 [22]. Although no confirmed cases of TBEV, OZV, or YezoV have been reported in Korea—and none of these viruses were detected in this study—their documented presence in neighboring countries suggests a potential risk of future introduction into Korea.

Among the 1035 samples analyzed in this study, 969 tested positive, resulting in an overall positivity rate of 93.6%. Detection rates for all seven pathogens were consistently higher with real-time PCR than with conventional PCR, likely reflecting the superior sensitivity of real-time PCR in identifying low concentrations of pathogen DNA. This finding aligns with previous reports demonstrating that real-time PCR generally offers higher detection sensitivity compared to conventional PCR [23].

Geographically, samples collected from northern regions exhibited uniformly high positivity rates, with average detection rates exceeding 95% across nearly all surveyed sites. These sampling locations were originally selected by the NIWDC based on regions with a high incidence of human SFTS cases. Anticipating elevated SFTSV infection rates in local tick populations, the NIWDC prioritized sample collection from Korean water deer in these high-risk northern areas. Given that ticks are vectors not only of SFTSV but also of diverse TBD pathogens—and that Korean water deer are widely distributed tick hosts in Korea [5]—these animals were hypothesized to serve as important reservoirs for multiple TBDs. The consistently high positivity rates observed in deer from northern regions support this hypothesis and correspond with known regional distributions of human SFTS cases and presumed tick infection prevalence.

Seasonally, Korean water deer are distributed nationwide throughout the year and are recognized as major hosts for ticks. In this study, individuals captured in autumn were frequently found to carry heavy tick burdens. This observation is consistent with prior studies indicating a seasonal increase in tick larvae during autumn, contributing to a rise in overall tick density during this period [24]. These findings suggest a heightened likelihood of tick exposure among Korean water deer in autumn. Although tick-borne pathogens were detected in Korean water deer throughout the year, both tick burden and pathogen positivity rates peaked during the autumn months, indicating an elevated seasonal risk of transmission. This pattern is supported by monthly prevalence data, which showed the highest overall positivity rates in September (98.1%) and October (99.2%). Notably, *A. phagocytophilum* reached 84.1% in September and 89.2% in October. These results highlight autumn as a critical period for both tick activity and the transmission of tick-borne pathogens in Korean water deer.

Several *Anaplasma* species were detected in this study, highlighting the need for continued TBD surveillance in Korea. Notably, some of these pathogens remain poorly characterized with respect to their ecological and geographic distributions in wildlife hosts. Currently, only *A. phagocytophilum*, *A. capra*, *A. ovis*, and *A. platys* are recognized as zoonotic among the *Anaplasma* genus [25,26,27]. Of particular concern, *A. phagocytophilum* is a well-documented zoonotic agent that targets granulocytic white blood cells in mammalian hosts, causing HGA [28]. This pathogen has been identified in multiple tick species, and there is increasing concern about its potential emergence and transmission dynamics within Korea [29]. Supporting its established presence, *A. phagocytophilum* was previously detected in 18 out of 42 (42.9%) wild Korean water deer sampled in Gyeongbuk and Gangwon Provinces [30]. In the present study, this pathogen was identified in 77.7% of samples—the highest positivity rate among all detected TBD pathogens—providing compelling evidence of widespread systemic infection in Korean water deer.

*Anaplasma bovis* also represents a significant TBD pathogen. First identified in cattle in Brazil in 1936, it is now distributed throughout Asia, Africa, the Americas, and southern Europe [31]. Recent reports of human infections in China [32] and the United States [33] suggest an expanding geographic range and growing relevance to public health. In Korea, *A. bovis* has been detected in 4 of 173 tick pools (2.3%) collected from Korean water deer between 2013 and 2017 [14] and in 5 of 42 (11.9%) wild deer samples [30], indicating its presence in both vectors and hosts. In this study, *A. bovis* was detected in 2.5% of samples, reinforcing evidence of systemic infection in wild cervids and underscoring the need for wildlife-based surveillance to better understand its zoonotic potential.

*Anaplasma capra*, first identified in goats and humans with a history of tick exposure in China in 2015, was later confirmed as a novel species through phylogenetic analysis [27]. In Korea, *A. capra* was detected in 5 of 173 tick pools (2.9%) from Korean water deer collected between 2013 and 2017 [14], suggesting its circulation within cervid–tick ecological networks. A separate study reported its presence in 35 of 198 samples (17.8%) from Korean water deer collected between 2015 and 2018 [34], providing further evidence of systemic infection in this species. Given that *A. phagocytophilum* is a recognized zoonotic pathogen and *A. capra* has also been implicated in human infections in China, both species warrant close monitoring from a public health standpoint. In this study, *A. capra* was identified in 0.8% of samples, further supporting its potential infectivity in mammalian hosts and emphasizing the need for further investigation into its transmission dynamics.

Strains genetically similar to *A. phagocytophilum*, referred to in this study as APLA, have been identified in a variety of tick species parasitizing ruminants in Japan [35], including both cattle [36] and sika deer [37]. Phylogenetic analyses have demonstrated that these strains constitute a distinct monophyletic clade closely related to *A. phagocytophilum* yet exhibiting clear genetic divergence [38]. Their association with non-canonical tick vectors and the lack of clinical manifestations in infected animals further support their classification as novel *Anaplasma* strains, initially described in Japan [38]. In Korea, *A. phagocytophilum*-like strains have also been detected in domestic animals and ticks. For example, APLA was previously identified in 2.6% of cattle and was characterized by sequence divergence in the *groEL* gene [39], while APLB—a strain first reported in China—has been found in questing ticks from various regions of Korea [40]. However, to date, neither APLA nor APLB has been reported in Korean water deer or in any other cervid species globally. In the present study, APLA and APLB were detected in 0.9% and 5.7% of samples, respectively, representing the first global detection of these strains in this host species. These findings substantially broaden the recognized wildlife host range for both strains and underscore the importance of wildlife-based surveillance in elucidating their ecological roles and potential zoonotic risk.

*Borrelia* species represent another group of tick-borne pathogens that pose considerable health risks to both animals and humans. These spirochetes are generally categorized into two major groups: those associated with Lyme disease and those causing relapsing fever [41]. Lyme disease-associated *Borrelia* species, such as *B. burgdorferi* and *B. afzelii*, are predominantly transmitted by hard ticks. In Korea, *B. afzelii* was molecularly detected in ticks collected from wild Korean water deer between 2013 and 2015 [13]. The relapsing fever group has recently undergone further classification based on vector type, including soft-tick-borne species such as *B. duttonii* and hard-tick-borne species such as *B. theileri* [42]. In Korea, *B. theileri* has been reported in various hosts and vectors, including raccoon dogs [43], indigenous cattle [44], and ticks [45], suggesting a broader host and geographic distribution than previously recognized. The transmission dynamics of *Borrelia* spp., including *B. theileri*, are shaped not only by tick abundance but also by host–vector interactions and environmental factors. Previous Korean studies have documented a temporal lag between the peak of tick activity and the incidence of human disease. For instance, the detection of *Borrelia* spp. in ticks peaked in July, while human Lyme disease cases peaked in August [46]. Similarly, in a 2020 study, SFTSV detection in ticks peaked in August, whereas human cases peaked in October [24]. In our study, the highest *B. theileri* positivity was observed in October, potentially reflecting a similar temporal lag. Increased tick activity during the autumn season may lead to more frequent host–vector interactions, contributing to a delayed rise in pathogen detection. To our knowledge, this is the first global report of *B. theileri* infection in Korean water deer. These findings emphasize the role of complex host–vector–environment interactions in shaping the epidemiology of tick-borne pathogens and highlight the need for further investigation into their transmission cycles.

In our study, both *Babesia* and *Theileria* spp. tested positive by real-time PCR. However, only *Theileria* spp. were detected using conventional PCR, which identified three species: *T. capreoli*, *T. cervi*, and *T. luwenshuni*. This discrepancy may be attributable to cross-reactivity with *Theileria* DNA, given the high sequence similarity of the 18S rRNA gene between *Babesia* and *Theileria*, even when TaqMan probes are employed [47]. A pertinent example is the reclassification of *Babesia equi* as *Theileria equi*, based on phylogenetic analysis of the 18S rRNA gene in conjunction with morphological characteristics [48]. Therefore, the observed *Babesia* signal in this study may reflect cross-reactivity with *Theileria* spp., particularly *T. capreoli*, which exhibits high sequence homology with *Babesia capreoli* within the 18S rRNA region. To enhance species-level discrimination, the sequencing of real-time PCR amplicons or the use of alternative genetic markers with greater discriminatory power is recommended.

*Theileria* is a clinically and economically significant tick-borne protozoan parasite that predominantly targets the bloodstream and presents a major threat to the global livestock industry [49]. In addition to domestic animals, *Theileria* spp. have also been detected in wild ruminants, including roe deer (*Capreolus capreolus* L.), in which *T. capreoli* has been previously identified [50]. In Korea, *T. capreoli* was first reported in Korean water deer, with a prevalence of 5.6% (1/18) during the 2008–2009 period [51], and more recently in ticks at a detection rate of 25% (4/16) in 2024 [46]. However, the limited sample sizes in these studies constrain the broader applicability of their findings. In contrast, our investigation identified *T. capreoli* as the third most prevalent pathogen, with a positivity rate of 27.4%, underscoring the need for further research into its epidemiological significance and its potential impact on wildlife health and vector ecology.

Among *Theileria* species, *T. cervi* is recognized as a causative agent of cervine theileriosis [52], whereas the closely related *Theileria* sp. Thrivae has been identified as a non-pathogenic strain in sika deer in Japan [53]. A genetically similar *Theileria* sp. was also reported in China and designated as *T. cervi* [49]. Of note, *T. cervi* identified in white-tailed deer and elk (*Cervus canadensis*) in the United States and Canada [54] is genetically distinct from both *Theileria* sp. Thrivae and the *T. cervi* strains reported in Japan and China. Phylogenetic analysis of the *T. cervi* sequences obtained in this study demonstrated clustering with a previously reported Chinese strain (KT959224), suggesting that the isolate identified herein is genetically distinct from North American strains. In our cohort, *T. cervi* was the least prevalent of the three *Theileria* species, with a detection rate of 0.6%.

*Theileria luwenshuni* was initially believed to be confined to sheep and goats in China [55]. However, more recent evidence indicates its pathogenic potential, particularly in association with high mortality among small ruminants [56]. In Korea, *T. luwenshuni* has been detected in all 23 wild roe deer examined on Jeju Island, as well as in 34.8% of ticks collected from these animals [57]. A 2015 study also reported its presence in 10 out of 18 Korean water deer (55.6%) [58]. Consistent with these findings, *T. luwenshuni* was detected in 60.7% of samples in our study, making it the second most prevalent pathogen among all tick-borne agents identified. These data support the conclusion that *T. luwenshuni* is the most widely distributed *Theileria* species among wild cervids in Korea and may play a predominant role in the regional epidemiology of *Theileria* infections in these hosts.

Although this study focused on the molecular detection of TBDs in Korean water deer, it did not include the collection or analysis of ticks parasitizing the hosts. A previous study by our group has provided baseline information on ticks parasitizing Korean water deer, including the detection of multiple pathogens [14]. Given that tick burden can influence pathogen transmission dynamics, future studies should incorporate data on tick species composition and infestation levels.

## 5. Conclusions

This study presents comprehensive molecular evidence of multiple tick-borne pathogens in Korean water deer, including the first global identification of APLA, APLB, and *B. theileri* in this host species. The notably high prevalence of *T. luwenshuni* and *A. phagocytophilum* suggests that Korean water deer may serve as significant reservoir hosts for tick-borne diseases in Korea. These findings underscore the critical importance of wildlife-based surveillance in elucidating the ecological dynamics and zoonotic potential of emerging tick-borne pathogens. Further investigation is warranted to delineate transmission pathways, evaluate associated public health risks, and guide the development of targeted vector control strategies.

## Figures and Tables

**Table 1:** Pathogens detected as positive by real-time PCR in 1035 clinical samples.

Pathogen	Positive Samples (%)
*Anaplasma* spp.	937 (90.5; CI 88.7–92.3)
*Babesia* spp.	325 (31.4; CI 28.6–34.2)
*Borrelia* spp.	173 (16.7; CI 14.4–19.0)
*Theileria* spp.	965 (93.2; CI 91.7–94.8)
Tick-borne encephalitis virus	0
OZ virus	0
Yezo virus	0

Abbreviations: CI, confidence intervals.

**Table 2:** Geographic distribution of infections identified using conventional PCR.

Area	Region		No. of Tested	No. Positive (%)									
				*Anaplasma phagocytophilum*	APLA	APLB	*Anaplasma bovis*	*Anaplasma capra*	*Borrelia theileri*	*Theileria capreoli*	*Theileria cervi*	*Theileria luwenshuni*	Total
Northern	Gangwon	Wonju	134	108 (80.6; CI 73.9–87.3)	0	7 (5.2; CI 1.5–9.0)	3 (2.2; CI 0–4.7)	0	10 (7.5; CI 3.0–11.9)	40 (29.9; CI 22.1–37.6)	1 (0.7; CI 0–2.2)	82 (61.2; CI 52.9–69.4)	128 (95.5; CI 92.0–99.0)
		Chuncheon	72	49 (68.1; CI 57.3–78.8)	3 (4.2; CI 0–8.8)	4 (5.6; CI 0.3–10.8)	2 (2.8; CI 0–6.6)	4 (5.6; CI 0.3–10.8) *	3 (4.2; CI 0–8.8)	14 (19.4; CI 10.3–28.6)	0	53 (73.6; CI 63.4–83.8) *	70 (97.2; CI 93.4–100)
	Gyeonggi	Gapyeong	60	53 (88.3; CI 80.2–96.5)	1 (1.7; CI 0–4.9)	4 (6.7; CI 0.4–13.0)	0	0	2 (3.3; CI 0–7.9)	19 (31.7; CI 19.9–43.4)	0	40 (66.7; CI 54.7–78.6)	59 (98.3; CI 95.1–100)
		Namyangju	56	50 (89.3; CI 81.2–97.4) *	2 (3.6; CI 0–8.4)	3 (5.4; CI 0–11.3)	0	1 (1.8; CI 0–5.3)	5 (8.9; CI 1.5–16.4)	23 (41.1; CI 28.2–54.0)	3 (5.4; CI 0–11.3) *	30 (53.6; CI 40.5–66.6)	56 (100) *
		Yangpyeong	31	23 (74.2; CI 58.8–89.6)	0	5 (16.1; CI 3.2–29.1)	1 (3.2; CI 0–9.4)	0	2 (6.5; CI 0–15.1)	17 (54.8; CI 37.3–72.4) *	0	14 (45.2; CI 27.6–62.7)	31 (100) *
		Yeoncheon	60	40 (66.7; CI 54.7–78.6)	0	2 (3.3; CI 0–7.9)	0	1 (1.7; CI 0–4.9)	2 (3.3; CI 0–7.9)	14 (23.3; CI 12.6–34.0)	1 (1.7; CI 0–4.9)	30 (50.0; CI 37.3–62.7)	46 (76.7; CI 66.0–87.4)
		Pocheon	85	72 (84.7; CI 77.1–92.4)	0	7 (8.2; CI 2.4–14.1)	0	0	5 (5.9; CI 0.9–10.9)	37 (43.5; CI 33.0–54.1)	0	45 (52.9; CI 42.3–63.6)	82 (96.5; CI 92.5–100)
Central	Gyeongbuk	Andong	125	96 (76.8; CI 69.4–84.2)	1 (0.8; CI 0–2.4)	6 (4.8; CI 1.1–8.5)	5 (4.0; CI 0.6–7.4)	0	9 (7.2; CI 2.7–11.7)	17 (13.6; CI 7.6–19.6)	1 (0.8; CI 0–2.4)	86 (68.8; CI 60.7–76.9)	117 (93.6; CI 89.3–97.9)
		Yeongyang	78	48 (61.5; CI 50.7–72.3)	1 (1.3; CI 0–3.8)	4 (5.1; CI 0.2–10.0)	2 (2.6; CI 0–6.1)	0	7 (9.0; CI 2.6–15.3)	10 (12.8; CI 5.4–20.2)	0	43 (55.1; CI 44.1–66.2)	59 (75.6; CI 66.1–85.2)
	Chungnam	Gongju	104	75 (72.1; CI 63.5–80.7)	0	6 (5.8; CI 1.3–10.3)	4 (3.8; CI 0.2–7.5)	0	8 (7.7; CI 2.6–12.8)	27 (26.0; CI 17.5–34.4)	0	60 (57.7; CI 48.2–67.2)	95 (91.3; CI 85.9–96.7)
Southern	Gyeongnam	Jinju	115	96 (83.5; CI 76.7–90.3)	0	4 (3.5; CI 0.1–6.8)	3 (2.6; CI 0–5.5)	2 (1.7; CI 0–4.1)	9 (7.8; CI 2.9–12.7)	33 (28.7; CI 20.4–37.0)	0	76 (66.1; CI 57.4–74.7)	114 (99.1; CI 97.4–100)
		Hapcheon	115	94 (81.7; CI 74.7–88.8)	1 (0.9; CI 0–2.6)	7 (6.1; CI 1.7–10.5)	6 (5.2; CI 1.2–9.3)	0	7 (6.1; CI 1.7–10.5)	33 (28.7; CI 20.4–37.0)	0	69 (60.0; CI 51.0–69.0)	112 (97.4; CI 94.5–100)
Total			1035	804 (77.7; CI 75.1–80.2)	9 (0.9; CI 0.3–1.4)	59 (5.7; CI 4.3–7.1)	26 (2.5; CI 1.6–3.5)	8 (0.8; CI 0.2–1.3)	69 (6.7; CI 5.1–8.2)	284 (27.4; CI 24.7–30.2)	6 (0.6; CI 0.1–1.0)	628 (60.7; CI 57.7–63.7)	969 (93.6; CI 92.1–95.1)

Abbreviations: APLA, *Anaplasma phagocytophilum*-like A; APLB, *Anaplasma phagocytophilum*-like B; CI, confidence intervals. * Significant differences in prevalence (*p* < 0.05).

**Table 3:** Temporal distribution of infections identified using conventional PCR.

Collection Month	No. of Tested	No. Positive (%)									
		*Anaplasma phagocytophilum*	APLA	APLB	*Anaplasma bovis*	*Anaplasma capra*	*Borrelia theileri*	*Theileria capreoli*	*Theileria cervi*	*Theileria luwenshuni*	Total
April	56	44 (78.6; CI 67.8–89.3)	0	0	3 (5.4; CI 0–11.3)	0	5 (8.9; CI 1.5–16.4)	10 (17.9; CI 7.8–27.9)	0	27 (48.2; CI 35.1–61.3)	48 (85.7; CI 76.5–94.9)
May	69	50 (72.5; CI 61.9–83.0)	0	5 (7.2; CI 1.1–13.4)	3 (4.3; CI 0–9.2)	0	3 (4.3; CI 0–9.2)	1 (1.4; CI 0–4.3)	0	50 (72.5; CI 61.9–83.0)	64 (92.8; CI 86.6–98.9)
June	83	64 (77.1; CI 68.1–86.1)	0	2 (2.4; CI 0–5.7)	5 (6.0; CI 0.9–11.1) *	1 (1.2; CI 0–3.6)	5 (6.0; CI 0.9–11.1)	5 (6.0; CI 0.9–11.1)	0	70 (84.3; CI 76.5–92.2) *	78 (94.0; CI 88.9–99.1)
July	89	57 (64.0; CI 54.1–74.0)	1 (1.1; CI 0–3.3)	2 (2.2; CI 0–5.3)	1 (1.1; CI 0–3.3)	4 (4.5; CI 0.2–8.8) *	5 (5.6; CI 0.8–10.4)	5 (5.6; CI 0.8–10.4)	2 (2.2; CI 0–5.3)	70 (78.7; CI 70.1–87.2)	86 (96.6; CI 92.9–100)
August	114	75 (65.8; CI 57.1–74.5)	0	2 (1.8; CI 0–4.2)	5 (4.4; CI 0.6–8.1)	2 (1.8; CI 0–4.2)	3 (2.6; CI 0–5.6)	19 (16.7; CI 9.8–23.5)	0	74 (64.9; CI 56.2–73.7)	94 (82.5; CI 75.5–89.4)
September	207	174 (84.1; CI 79.1–89.0)	3 (1.4; CI 0–3.1)	8 (3.9; CI 1.2–6.5)	7 (3.4; CI 0.9–5.8)	1 (0.5; CI 0–1.4)	9 (4.3; CI 1.6–7.1)	64 (30.9; CI 24.6–37.2)	0	131 (63.3; CI 56.7–69.9)	203 (98.1; CI 96.2–99.9)
October	250	223 (89.2; CI 85.4–93.0) *	4 (1.6; CI 0–3.2)	15 (6.0; CI 3.1–8.9)	2 (0.8; CI 0–1.9)	0	25 (10.0; CI 6.3–13.7)	99 (39.6; CI 33.5–45.7)	4 (1.6; CI 0–3.2)	139 (55.6; CI 49.4–61.8)	248 (99.2; CI 98.1–100) *
November	167	117 (70.1; CI 63.1–77.0)	1 (0.6; CI 0–1.8)	25 (15.0; CI 9.6–20.4) *	0	0	14 (8.4; CI 4.2–12.6)	81 (48.5; CI 40.9–56.1) *	0	67 (40.1; CI 32.7–47.6)	148 (88.6; CI 83.8–93.4)
Total	1035	804 (77.7; CI 75.1–80.2)	9 (0.9; CI 0.3–1.4)	59 (5.7; CI 4.3–7.1)	26 (2.5; CI 1.6–3.5)	8 (0.8; CI 0.2–1.3)	69 (6.7; CI 5.1–8.2)	284 (27.4; CI 24.7–30.2)	6 (0.6; CI 0.1–1.0)	628 (60.7; CI 57.7–63.7)	969 (93.6; CI 92.1–95.1)

Abbreviations: APLA, *Anaplasma phagocytophilum*-like A; APLB, *Anaplasma phagocytophilum*-like B; CI, confidence intervals. * Significant differences in prevalence (*p* < 0.05).

## Data Availability

Data supporting the conclusions of this article are included within the article. The newly generated sequences were submitted to the GenBank database under the accession numbers PV362643–PV362687, PV389464–PV389471, and PV436601–PV436637. The datasets used and/or analyzed during the present study are available from the corresponding author upon reasonable request.

## Supplementary Materials

The following supporting information can be downloaded at: https://www.mdpi.com/article/10.3390/ani15101499/s1, Table S1: GPS coordinates and collection dates of 1035 Korean water deer sampled between April and November 2023.

## Appendix A

Modified DNA and RNA Extraction Protocol for Korean Water Deer Spleen Tissue

(Adapted from the Clear-S™ Quick DNA Extraction Kit, InVirusTech)

This protocol was specifically optimized for the simultaneous extraction of DNA and RNA from the spleen tissue of Korean water deer. Please note that the procedure deviates from the standard Clear-S™ tissue extraction manual.

Pre-experiment Preparation:

1. Prepare 1.5 mL microcentrifuge tubes and add β-mercaptoethanol (reducing agent) at a ratio of 10 μL per 700 μL of DLD buffer (i.e., 10 μL + 700 μL per sample).
2. Add 45 mL of 100% ethanol to the WA buffer and 66 mL to the wash buffer as instructed by the kit.
3. Preheat the elution buffer (EB) to 37 °C prior to use.
4. Protocol Steps.
5. Add 710 μL of prepared lysis buffer (700 μL DLD + 10 μL β-mercaptoethanol) to ~10 mg of spleen tissue in a homogenization tube.
6. Homogenize using a tissue grinder at 3500 rpm for 30 s, pause for 10 s, and repeat twice. If homogenization is incomplete, repeat up to 1–2 additional cycles.
7. Vortex thoroughly and incubate at room temperature for 5–10 min. Then, centrifuge at 13,000 rpm for 1 min.
8. Label a pre-treatment column and collection tube. Transfer the supernatant (~710 μL) to the column and centrifuge at 13,000 rpm for 1 min.
9. Discard the upper spin column. To the remaining flow-through (~710 μL), add 710 μL of 100% ethanol (final volume: 1420 μL).
10. Attach a binding column to a new collection tube. Mix the ethanol-added lysate by pipetting 3–4 times.
11. Load 710 μL of the solution into the column and centrifuge at 13,000 rpm for 1 min. Discard the flow-through and wipe the tube rim.
12. Load the remaining 710 μL of the lysate and repeat the centrifugation step.
13. Add 700 μL of WA buffer, centrifuge at 13,000 rpm for 1 min, discard the flow-through, and reattach the column.
14. Add 500 μL of wash buffer, centrifuge at 13,000 rpm for 1 min, discard the flow-through, and repeat with another 500 μL followed by centrifugation for 3 min.
15. Transfer the column to a clean 1.5 mL tube. Add 200 μL of preheated EB buffer (37 °C) to the column and incubate at room temperature for 5 min.
16. Centrifuge at 13,000 rpm for 1 min to collect the final eluate.
17. Store nucleic acid extracts at –70 °C until analysis.
